# Retrograde intramedullary nail fixation with oblique fixed angle screws versus locking plates in periprosthetic supracondylar fractures after total knee arthroplasty

**DOI:** 10.1007/s00068-024-02530-x

**Published:** 2024-05-28

**Authors:** Franziska Rudolph, Alexander G. Brand, Georg Osterhoff, Christian Kleber, Andreas Roth, Johannes K. M. Fakler

**Affiliations:** 1 Department of Orthopedic, Trauma and Plastic Surgery, University Hospital of Leipzig, Liebigstr. 20, 04103, Leipzig, Germany; 2 Present Address: Department of Trauma-, Hand-, Reconstructive- and Spine Surgery, Hospital of Passau, Innstraße 76, 94032 Passau, Germany; 3 Present Address: Department of Traumatology, University Hospital of Zurich, Rämistrasse 100, 8091, Zurich, Switzerland

**Keywords:** Periprosthetic supracondylar fracture, Retrograde intramedullary nail, Locking plate, Complication, Functional outcome, Total knee arthroplasty

## Abstract

**Purpose:**

Common surgical procedures in the treatment of periprosthetic distal femur fractures (PPFF) include osteosynthesis with fixed angle locking plates (LP) and retrograde intramedullary nails (RIN). This study aimed to compare LPs to RINs with oblique fixed angle screws in terms of complications, radiographic results and functional outcome.

**Methods:**

63 PPFF in 59 patients who underwent treatment in between 2009 and 2020 were included and retrospectively reviewed. The anatomic lateral and posterior distal femoral angle (aLDFA and aPDFA) were measured on post-surgery radiographs. The Fracture Mobility Score (FMS) pre- and post-surgery, information about perceived instability in the operated leg and the level of pain were obtained via a questionnaire and previous follow-up (FU) examinations in 30 patients (32 fractures).

**Results:**

The collective (median age: 78 years) included 22 fractures treated with a RIN and 41 fractures fixed with a LP. There was no difference in the occurrence of complications (median FU: 21.5 months) however the rate of implant failures requiring an implant replacement was higher in fractures treated with a LP (*p* = 0.043). The aPDFA was greater in fractures treated with a RIN (*p* = 0.04). The functional outcome was comparable between both groups (median FU: 24.5 months) with a lower outcome in the post-surgery FMS (*p* =  < 0.001).

**Conclusion:**

Fractures treated with RIN resulted in an increased recurvation of the femur however the rate of complications and the functional outcome were comparable between the groups. The need for implant replacements following complications was higher in the LP group.

## Introduction

The number of performed total knee arthroplasties (TKAs) is constantly increasing [1]. Periprosthetic fractures of the distal femur (PPFF) are a challenging complication following primary or revision TKA occurring in 0.3% to 2.5% after primary TKA and even more after revision TKA [2–5]. Factors predisposing to PPFF apart from revision TKA are female gender, increased age [2, 4], osteoporosis or osteopenia and steroid therapy [5]. In addition, studies have shown that primary TKA itself leads to a significant decrease of bone mineral density in the first 6 months after surgery lasting up to 24 months, subsequently increasing the risk of periprosthetic fractures [6].

There are several options to manage PPFF, however the best way of treatment is discussed controversially [7]. Even though retrograde intramedullary nail fixation (RIN) and locking plate fixation (LP) have shown favorable results [8–10] and should be preferred over non-locking plate fixation [11] both treatment options face limitations. Retrograde intramedullary nailing requires compatibility with the femoral component of the prothesis as a closed or narrow intercondylar box, a femoral stem or implants proximal to the knee make passage of the nail difficult or impossible [12–14]. Fractures treated with internal fixation show varying ranges of complication, union and non-union rates in the literature [15–20] so that superiority of either RIN or LP is difficult to assess.

The objective of this study was to compare RIN with oblique fixed angle screws to fracture fixation via LP to determine advantages in clinical and functional outcome. In particular, mainly complex fractures classified as Su type II and III are presented in this study which have not been often analysed comparing these two methods. Outcome parameters included complications, the radiological outcome as well as the functional outcome regarding pain, subjective instability and mobility evaluated by the Fracture mobility score (FMS).

## Materials and methods

This single center retrospective cohort study is approved by the local ethics committee of the University of Leipzig (IN: 409/20-ek) and was performed in line with the principles of the 1964 Helsinki Declaration and its later amendments or comparable ethical standards. Informed consent was not required because of the retrospective design of the study and the evaluation of pre-existing medical records. All patients who agreed to participate in the follow-up questionnaire gave informed consent. The study is registered with the ResearchRegistry and the unique identifying number (UIN) is: researchregistry6813.

All patients aged 18 years or older with a cruciate retaining (CR) or posterior stabilized (PS) total knee replacement sustaining a supracondylar femoral fracture treated with either a RIN or a LP between 2009 and 2020 were included. The minimum follow-up was 6 months. Patients who died during the follow-up period without complications (3 patients) and patients with a shorter follow-up but complications (2 patients) were also included. In total, 59 patients with 63 fractures (4 bilateral) were identified.

In all patients, the implanted design was checked preoperatively in the surgical report of the knee replacement implantation. Intramedullary nail fixation was only attempted in open box designs. If it became apparent intraoperatively that the design did not permit intramedullary nail fixation, plate fixation was used.

The RIN featured two angle-stable oblique antero-posterior and optionally two angle-stable latero-medial blocking screws distally.

RINs were inserted through a minimal-invasive transpatellar approach. The polyethylene inlay had not to be changed or removed in any of the cases. In addition, a limited lateral approach for cerclage wiring was used in some cases. For LP a limited lateral approach was performed with percutaneous insertion of locking screws at the femoral shaft. Cerclage wiring was also used in some cases.

Exclusion criteria included any other method of surgery, pathological fractures (primary malignancy or metastatic), a fully constrained (hinged) TKA, intraoperative fractures or a loose TKA prior to the fracture event.

### Outcome measures

Clinical data was collected and reviewed using the clinic’s electronic database. Primary outcomes of the study involved the rate and the type of complications after surgical treatment. Complications were classified into hematoma/seroma, deep infection, peri-implant fracture and mechanical complication. Mechanical complications were defined as implant-, screw- or bolt-loosening, implant breakage, fracture displacement, delayed union or non-union.

Additionally, the necessity of re-osteosynthesis with an implant exchange or revision with a distal femoral replacement following complications was analyzed.

Demographic data containing the age, gender, Body-Mass-Index (BMI), comorbidities and the American Society of Anesthesiologists-Score (ASA-Score) was assessed next to laboratory parameters (c-reactive protein (CRP), hemoglobin, prothrombin time) on the day of admission. Furthermore, fracture and treatment characteristics including the fracture side, type of prothesis, type of fracture, primary external stabilization, presence of an ipsilateral proximal implant, inpatient days and operation time were recorded.

Radiographs taken directly post-surgery were retrospectively evaluated via the clinic’s radiographic database. Fractures were classified by two authors of the study according to the Su classification as well as the Lewis—Rorabeck classification [21, 22].

**Fig. 1:**
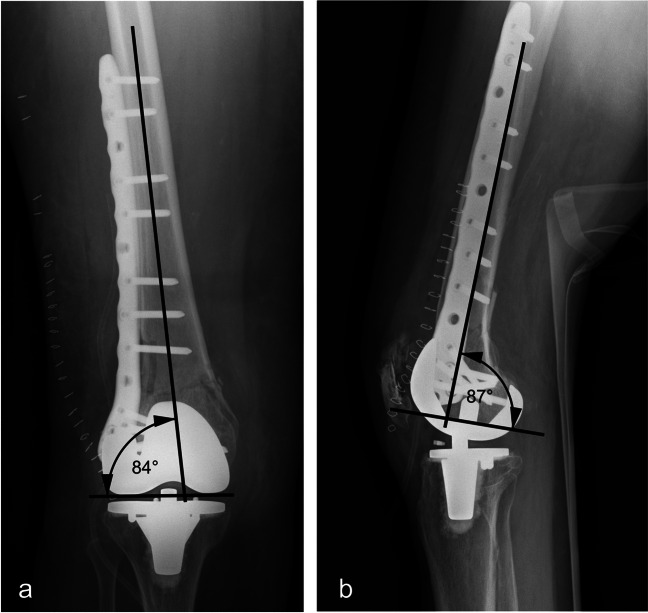
A.p. (**a**) and lateral (**b**) view radiographs showing the measuring methods for the aLDFA and aPDFA assessment

The anatomic lateral distal femoral angle (aLDFA) and the anatomic posterior distal femoral angle (aPDFA) were measured in anterior–posterior and lateral view radiographs to determine deviation in the femoral axis (Fig. 1).

Follow-up data was collected via the departments electronic database retrospectively from previous follow-up examinations or via a questionnaire. Variables assessed include the level of pain (numeric pain rating scale), subjective feeling of instability as well as the Fracture Mobility Score (FMS) pre- and post-surgery.

### Statistical analysis

Normal distribution of the data was verified using the Shapiro–Wilk test. An independent samples t-test was applied to detect significance in normally distributed data. Data with non-normal distribution and ordinal variables were compared by the Mann–Whitney-U-Test. Categorical parameters were analyzed using the X^2^- or Fisher’s exact Test according to sample size. The Wilcoxon signed-rank test was used to compare repeated measurements on a single sample. The alpha-level was set at 0.05 and all tests were two-sided. Statistical analysis was performed using IBM SPSS statistics version 27 (SPSS Inc., Chicago, Illinois).

## Results

**Fig. 2:**
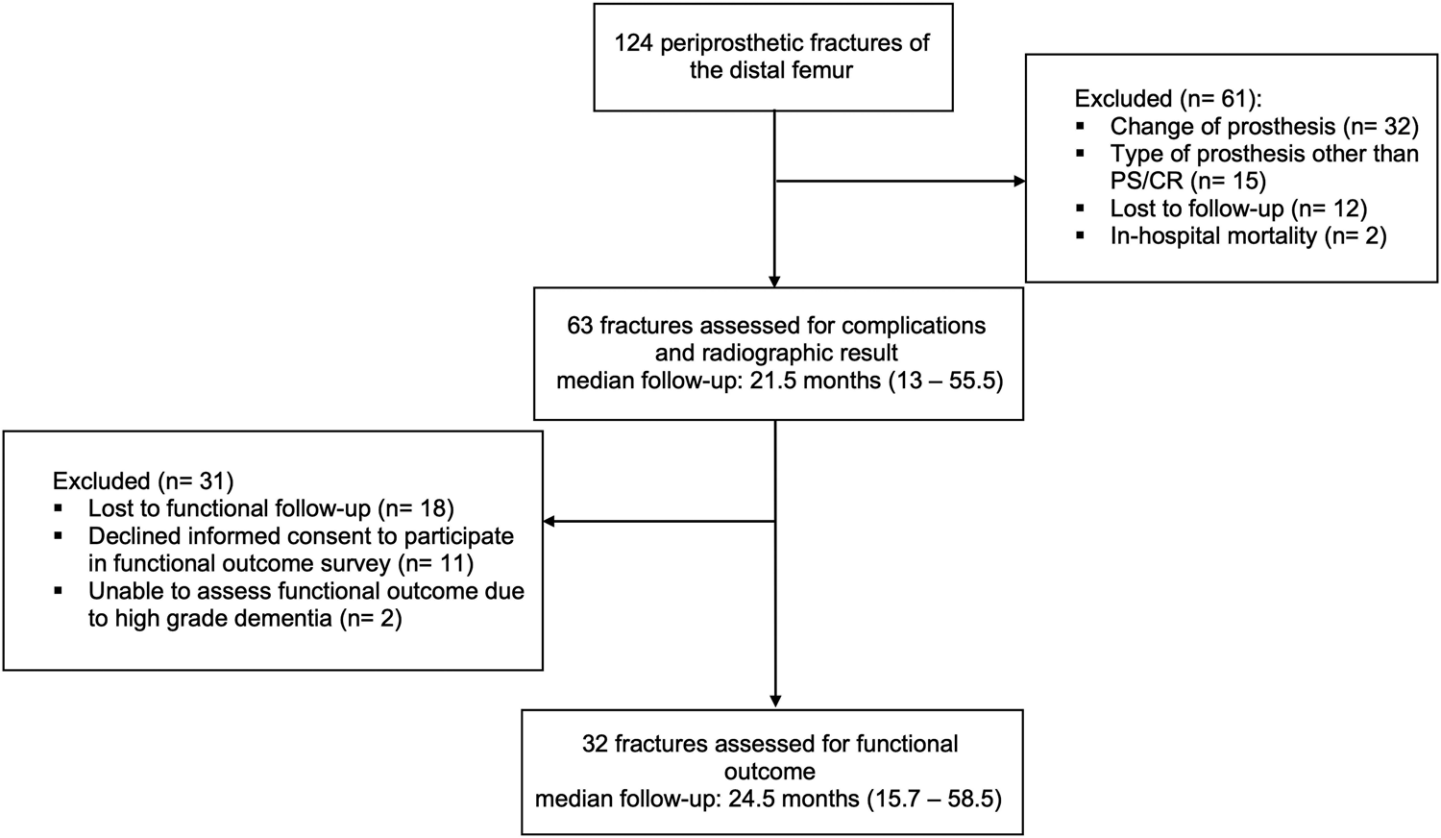
Flow Chart illustrating the process of fracture enrollment

59 patients with 63 fractures were finally enrolled in this study (Fig. 2). RIN was performed in 22 cases and LP was used in 41 cases.

The median time of follow-up for complications was 21.5 months (13 – 55.5). The collective included 15 male and 48 female knees with a median age of 78 years (72—86). The groups were comparable regarding age, BMI, ASA-Score, pre-operative laboratory parameters, type of prothesis and fracture related factors (Table 1). In addition, there was no difference in operation time (mean: 110.2 min ± 35.9) or inpatient days (median: 13d (10—26)) between the RIN and the LP cohort. The patient collective showed several comorbidities such as cardiovascular and kidney diseases, pulmonary dysfunction, and dementia. Patients with a RIN showed a significantly higher incidence of diabetes compared to patients treated with a LP (*p* = 0.012). The groups were comparable in fracture distribution and the majority of fractures were complex with 97% classified as Rorabeck II and 82.5% as Su type II or III (Table 1).

**Table 1:** Comparison of patient and fracture characteristics between the LP and the RIN group

Demographics		Intramedullary Nail (*n* = 22)	Locking Plate (*n* = 41)	*p* value
Gender	Male	6 (27.3%)	9 (22%)	0.636
	Female	16 (72.7%)	32 (78%)	
Age (y)		77 (66.0-86.5)	81 (73.5-86)	0.471
BMI		29 (26-34.3)	28 (25.5-31)	0.259
ASA-Score	I	2 (9%)	0 (0%)	0.490
	II	4 (18%)	15 (37%)	
	III	15 (68%)	26 (63%)	
	IV	1 (5%)	0 (0%)	
Comorbidities	cardiovascular	21 (95.5%)	35 (85%)	0.405
	Kidney disease	9 (41%)	8 (19.5%)	0.068
	Pulmonary dysfunction	3 (14%)	5 (12%)	1.000
	dementia	4 (18%)	9 (22%)	1.000
	diabetes	11 (50%)	8 (19.5%)	0.012
CRP (mg/l)		15.4 (2.2-47.3)	10.3 (2-44.3)	0.756
Hemoglobin (mmol/l)		6.7 (5.9-7.5)	6.8 (5.9-8)	0.444
Prothrombin time (%)		88 (78.8-98.5)	95 (84-102)	0.391
Fracture side	Left	11 (50%)	20 (49%)	0.926
	Right	11 (50%)	21 (51%)	
Type of prothesis	CR	21 (95.5%)	34 (83%)	0.243
	PS	1(4.5%)	7 (17%)	
Type of fracture	Open fracture	1 (4.5%)	1 (2%)	1.000
	Closed fracture	21 (95.5%)	40 (98%)	
Primary external stabilization		3 (14%)	3 (7%)	0.413
ipsilateral proximal implant		4 (18%)	14 (34%)	0.181
Su classification	I	5 (23%)	6 (15%)	0.496
	II	8 (36%)	21 (51%)	
	III	9 (41%)	14 (34%)	
Inpatient days (d)		15 (10.8-25)	13 (10-26)	0.431
Operation time (min)		108.2 (± 28.6)	111.4 (± 39.7)	0.741

Values are given as median (interquartile range), mean (± standard deviation) or absolute numbers (percentage). *BMI* body mass index (kg/m^2^), *ASA – Score* American society of anesthesiologists score, *CRP* C-reactive protein

### Complications and revision surgery

Two patients died in hospital after revision surgery shortly after the primary intervention due to a surgical site infection and a tumorous malignancy.

24 Patients (38%) sustained complications and in 22 cases (35%) surgical revision was needed. Multiple complications per patient were recorded in some cases. No significant differences were observed in the occurrence of nearly all various complications between the two groups (Table 2). The most common complications recorded were mechanical related including implant-, bolt- or screw-loosening, implant breakage, fracture displacement, delayed union or non-union. Deep infections in 7 patients (11%) accounted for the second most frequent complication (Table 2). Only considering Su type III fractures (*n* = 23) there was no significant difference in the occurrence of complications and the method of fixation (*p* = 1.000).

**Table 2:** Surgical complications and radiological outcome after surgery

	Intramedullary Nail (*n* = 22)	Locking Plate (*n* = 41)	*p* value
Surgical complications (overall)	7 (32%)	17 (42%)	0.452
Surgical complications requiring revision surgery	6 (27%)	16 (39%)	0.351
Hematoma/Seroma	1 (5%)	3 (7%)	1.000
Infection (deep)	1 (5%)	6 (15%)	0.405
Peri-implant-fracture	1 (5%)	0 (0%)	0.349
Mechanical complications	5 (23%)	10 (24%)	0.883
Implant failure requiring implant replacement	1 (5%)	11 (27%)	0.043
aLDFA (degree)	85 (82.8-88)	85 (82-87.5)	0.711
aPDFA (degree)	92.5 (88.8-100.5)	87 (84.5-94)	0.04

Values are given as median (interquartile range) or absolute numbers (percentage)

*aLDFA* anatomic lateral distal femoral angle, *aPDFA* anatomic posterior distal femoral angle

The necessity for an implant replacement with re-osteosynthesis or distal femoral replacement was significantly higher in patients treated with LP (Table 2). A detailed overview of all revised cases is given in Table 3.

**Table 3:** Detailed overview of revised cases with complications

Case	Implant	TKA type	Su classification	Complication type	Revision
1	LP	PS	II	peri-implant infection	LP and TKA removal; staged revision and DFR
2	LP	PS	III	varus malunion	LP removal, corrective osteotomy and re-osteosynthesis with RIN
3	LP	PS	I	secondary fracture displacement	LP removal and re-osteosynthesis with new LP, medial strut graft and cerclage
4	LP	CR	III	secondary fracture displacement	LP and TKA removal, arthrodesis
5	LP	CR	II	non-union, breakage of LP	LP removal, re-osteosynthesis with new LP
6	LP	CR	III	peri-implant infection	debridement and antibiotics
7	LP	CR	III	peri-implant infection	debridement and antibiotics
8	LP	CR	II	secondary fracture displacement	LP removal and re-osteosynthesis with new LP
9	LP	CR	II	infected hematoma	hematoma evacuation, debridement, antibiotics
10	LP	CR	II	non-union, breakage of LP	LP removal, re-osteosynthesis with new LP
11	LP	CR	III	hematoma	hematoma evacuation
12	LP	CR	II	secondary fracture displacement	LP and TKA removal and revision with DFR
13	LP	CR	II	secondary fracture displacement	LP removal, re-osteosynthesis with double-plating
14	LP	CR	II	screw pull out, secondary displacement, infection	LP and TKA removal, staged revision and DFR
15	LP	CR	II	varus malunion, knee instability	exchange of polyethylene inlay
16	LP	CR	II	peri-implant infection	LP removal, debridement, re-osteosynthesis with new LP
17	RIN	CR	III	malpositioning of blocking screws	exchange of distal screws
18	RIN	CR	I	secondary fracture displacement	additional short LP
19	RIN	CR	I	RIN malpositioning with polyethylene impingement of the distal end	RIN inserted more proximally, exchange of all blocking screws
20	RIN	CR	II	subtrochanteric peri-implant fracture at the proximal nail tip	dynamic hip screw with nail retention
21	RIN	CR	II	peri-implant infection	RIN and TKA removal; staged revision, DFR
22	RIN	CR	III	secondary retrograde protrusion of the nail	TKA revision with polyethylene adaption and nail retention

*RIN* retrograde intramedullary nail, *LP* locking plate, *TKA* total knee arthroplasty, *CR* cruciate retaining, *PS* posterior stabilized, *DFR* distal femoral replacement

### Functional outcome

Complete outcome parameters were available for 32 fractures and all patients included have a follow-up > 6 months. The median time of follow-up was 24.5 months (15.7—58.5). Regarding the subjective feeling of knee instability in everyday activities there was no significant difference between the two groups (*p* = 0.130). Five out of 20 patients (25%) in the LP group reported a feeling of instability whereas seven out of 12 patients (58%) in the RIN group felt unstable.

The score on the numeric pain rating scale (0–10) showed no difference in the level of pain between patients treated with a LP or RIN (Table 4).

**Table 4:** Pre-operative mobility and functional outcome parameters

	Intramedullary Nail (*n* = 12)	Locking Plate (*n* = 20)	*p* value
Feeling of instability	7 (58%)	5 (25%)	0.130
Pain (numeric pain rating scale 0–10)	3.5 (2.0-5.75)	3.0 (1.25-4.0)	0.397
Fracture Mobility Score (pre-surgery)*	*n* = 11	*n* = 17	
Freely mobile without aids	6 (55%)	10 (59%)	0.781
Mobile outdoors with one aid	1 (9%)	1 (6%)	
Mobile outdoors with two aids or frame	3 (27%)	6 (35%)	
Some indoor mobility but never going outside without help	1 (9%)	0 (0%)	
No functional mobility (no use of lower limbs e.g. wheelchair)	0 (0%)	0 (0%)	
Fracture Mobility Score (post-surgery)*	*n* = 11	*n* = 17	
Freely mobile without aids	2 (18%)	2 (12%)	0.458
Mobile outdoors with one aid	3 (27%)	3 (18%)	
Mobile outdoors with two aids or frame	3 (27%)	6 (35%)	
Some indoor mobility but never going outside without help	2 (18%)	3 (17.5%)	
No functional mobility (no use of lower limbs e.g. wheelchair)	1 (9%)	3 (17.5%)	

*Two patients with bilateral fractures were evaluated separately for the outcome of mobility. Values are given as median (interquartile range) and absolute numbers (percentage)

The level of mobility after surgery was assessed in 30 patients (32 fractures). There was no difference in the FMS pre-surgery and post-surgery between the LP and the RIN cohort (Table 4). However, the FMS for all patients was significantly lower after surgery (p < 0.001) showing a decrease in mobility. Two patients with bilateral fractures were reviewed separately. The fracture treatment was performed with a LP bilateral for one patient and a RIN and LP for fracture stabilization on the other patient. Both patients showed a lower outcome of mobility after surgery from walking with a frame pre-surgery to no functional mobility in a wheelchair post-surgery and from being fully mobile without a walking device to walking with one walking aid outside.

### Radiological outcome

No difference was found in femoral axis deviation resulting in varus or valgus position between the LP and the RIN group measuring the aLDFA on radiographs taken directly post-surgery. However, in lateral view imaging the RIN group showed a significant extension deformity of the femur with a median aPDFA of 92.5° (88.8—100.5) compared to the LP group with a median aPDFA of 87° (84.5 – 94; *p* = 0.04) (Table 2).

## Discussion

Choosing the right method of fixation remains a challenge for surgeons when treating periprosthetic supracondylar fractures as patient characteristics and prosthesis related factors need to be considered.

In this study, we found that the rate of overall surgical complications (42% vs. 32%; *p* = 0.452) as well as surgical complications requiring a revision (39% vs. 27%; *p* = 0.351) were comparable between LPs and RINs. These results are in contrast to the findings of Horneff et al. [23] presenting higher revision rates after RIN. Whereas 14 patients with a RIN needed to be revised in their study only four patients in the LP group required a revision (*p* = 0.05). Consistent with our findings are the results of Gondalia et al. and Aldrian et al. [24, 25] reporting similar rates of treatment related complications between RIN and LP.

**Fig. 3:**
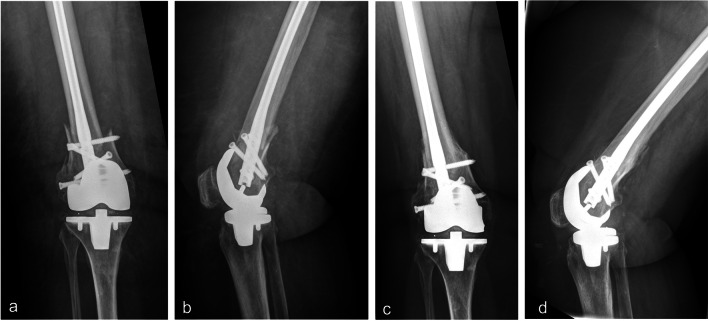
Initial post-operative a.p. (**a**) and lateral (**b**) view radiographs of a periprosthetic supracondylar fracture treated with a retrograde intramedullary nail with oblique fixed angle locking screws. Follow-up radiographs in a.p. (**c**) and lateral (**d**) view taken 7.5 months after fracture fixation showing fracture union and the formation of callus

Nevertheless, pre-operative patient characteristics should be analyzed when comparing complications and rates of revision. In the studies mentioned above, there is no further information on comorbidities or ASA-Scores of patients that may predispose to surgical complications. Even though we analyzed a significantly higher number of patients with diabetes in the RIN group (50% vs. 19.5%; *p* = 0.012) the incidence of complications was comparable to patients treated with a LP. Meneghini et al. [26] evaluated pre-existing comorbidities and the comparable groups showed an insignificant failure rate twice as high in patients treated with a LP compared to RIN fixation. However, they encourage the use of modern intramedullary nails with locked distal screws. This is consistent to our findings showing no difference in the occurrence of complications between LP and fracture fixation via a modern retrograde nail with oblique fixed angle locking screws. The separate evaluation of patients sustaining a low Su Type III fracture treated with RIN showed comparable results to patients with LP fixation. Consequently, implant design may also influence the incidence of complications and newly designed nails have made RIN a viable option for PPFF. Figure 3 shows the initial post-surgery and follow-up radiographs of a PPFF treated with a modern RIN with oblique fixed angle fixation possibilities.

Our study cohort shows an equal distribution of fracture location (*p* = 0.496) with a majority of complex Su type II (nail vs. plate: 36% vs. 51%) and III (nail vs. plate: 41% vs. 34%) fractures. This is in contrast to other studies [10, 25] excluding Su Type III fractures or not including Su Type III fractures treated with a RIN. Matlovich et al. [8] classified their fractures into high (above the TKA) and low fractures (at/below the TKA) and included a comparable number of low fractures in their study (nail vs. plate: 7 vs. 11). Consistent to our results they found no higher incidence of infections between the treatment groups. However, the overall re-operation rate was higher in patients treated with a RIN. This is contrary to our results with acceptable and comparable outcomes for RIN even in low Su Type III fractures below the TKA flange.

The rather high rate of revision surgeries (35%) in our study could be explained by the advanced age and various comorbidities of the study participants compared to other studies [23, 24]. Furthermore, as mentioned above a high number of very distal and complex fractures was treated. Additionally, since the rate of complications is the primary outcome of our study every occurred surgical complication was recorded. Other studies may define complications widely as delayed or non-union [9] and only record specific complications as the rate of complications is a secondary outcome.

Next to complications, this study evaluates the necessity for an implant replacement with re-osteosynthesis or distal femoral replacement implicating a more complex and invasive revision. A significantly higher rate of implant exchange was demonstrated for LP compared to RIN (5% vs. 27%; *p* = 0.043). This is explained by higher rates of plate breakages, tear-outs and infections requiring a replacement of the implant. Park et al. [27] indicate a possible advantage of double plating in the treatment of low PPFF as an additive medial LP increases fracture stability which might be superior to single LP. Complications in the RIN group included screw- or bolt-loosening as well as a peri-implant fracture, an infection and a secondary fracture displacement additionally stabilized with a LP. However, only one implant replacement was needed following these complications.

Patients treated with either a RIN or a LP were comparable in their pre- and post-operative mobility status. The authors decided to use the FMS as a tool to evaluate the mobility of patients as no universally validated scoring system for periprosthetic fractures exists. Even though the FMS is a score developed to assess the outcome of mobility in hip fracture patients it is validated and comparable to the Parker Mobility Score (PMS) [28]. Advantages can be seen in the simplicity of the score. Periprosthetic supracondylar fractures mainly affect the elderly so that complex scoring systems can be overwhelming for patients and are often not adapted to the circumstances of the patient. We suggest that the level of a patient’s autonomy, mainly influenced by the grading of mobility, is an important criterion to evaluate the clinical outcome. The comparison of the FMS not only post- but also pre-surgery is a strength of our study and allows an accurate analysis of the outcome of mobility. The comparable post-operative outcome of mobility between our groups is similar with the findings described by Gausden et al. [10] stating a comparable post-surgery ambulatory status.

Although there was no significant difference in the experienced feeling of instability in the operated leg between groups, the number of patients with an instability was more than twice as high after RIN fixation compared to locked plating. Results found by Matlovich et al. [8] specify a significantly higher rate of instability in patients treated with a RIN.

The authors assume that this may be related to a possible damage of the posterior cruciate ligament during insertion of the femoral nail in CR prosthesis. However, information on the status of instability prior to surgery is missing so that a pre-existing instability, which is common after primary TKA [29], cannot be precluded. The treatment with RINs resulted in a significant extension deformity of the femur matching similar results described in previous studies [10, 30]. A possible association between femoral hyperextension after RIN and the occurrence of a subjective instability may be further investigated in following studies.

### Limitations

Major limitations of the study include the retrospective design lacking randomization and the unequal sample size of the compared groups. Furthermore, with a high number of lost to follow-up patients only smaller groups were available for the evaluation of the functional outcome. Additionally, the use of RINs was not consistent but increased over the study period and could not be controlled in the analysis.

Finally, pre-fracture radiographs were not available as well as radiographs of the contralateral knee. Subsequently, inter-individual variations of the aLDFA, especially after TKA, could not be assessed.

## Conclusions

The findings of this study show that not only LP fixation but also RIN with oblique fixed angle screws are a viable option for treatment of periprosthetic supracondylar fractures even in very distal fractures classified as Su type III. RIN and LP fixation both show comparable and good results regarding clinical and functional outcome. However, it also needs to be considered that RIN results in an increased recurvation of the femur may causing an increased instability of the knee. Randomized controlled trials will be needed in the future to support these findings and provide further information on how to manage these complex fractures.

## Data Availability

Data and materials supporting the findings of this study are available from the corresponding author upon reasonable request.
